# Influencers in Policy Fields on Social Media: Global Longitudinal Study of Dietary Sodium Reduction Posts, 2006-2022

**DOI:** 10.2196/54506

**Published:** 2024-12-30

**Authors:** Alana Montoya, Lingchao Mao, Adam Drewnowski, Joshua Chen, Ella Shi, Aileen Liang, Bryan J Weiner, Yanfang Su

**Affiliations:** 1 Information School University of Washington Seattle, WA United States; 2 School of Industrial and Systems Engineering Georgia Institute of Technology Atlanta, GA United States; 3 Department of Epidemiology University of Washington Seattle, WA United States; 4 Skyline High School Sammamish, WA United States; 5 Redmond High School Redmond, WA United States; 6 Interlake High School Bellevue, WA United States; 7 Department of Global Health University of Washington Seattle, WA United States; 8 Evans School of Public Policy and Governance University of Washington Seattle, WA United States

**Keywords:** policy field, sodium intake, sodium consumption, cardiovascular disease, social media, health education, health promotion, dissemination, influence, Twitter, X, activity, priority, originality, popularity

## Abstract

**Background:**

Excessive sodium intake is a major concern for global public health. Despite multiple dietary guidelines, population sodium intakes are above recommended levels. Lack of health literacy could be one contributing issue and contemporary health literacy is largely shaped by social media.

**Objective:**

This study aims to quantify the posting behaviors and influence patterns on dietary sodium–related content by influencers in the policy field on X (formerly Twitter) across time.

**Methods:**

We first identified X users with a scope of work related to dietary sodium and retrieved their posts (formerly Tweets) from 2006 to 2022. Users were categorized into the policy groups of outer-setting organization, inner-setting organization, or individual, based on their role in the conceptual policy field. Network analysis was used to analyze interactions among users and identify the top influencers in each policy group. A 4D influence framework was applied to measure the overall influence, activity, priority, originality, and popularity scores. These measures were used to reveal the user-level, group-level, and temporal patterns of sodium-related influence.

**Results:**

We identified 78 users with content related to dietary sodium, with 1,099,605 posts in total and 14,732 dietary sodium posts. There was an increasing volume of sodium posts from 2010 to 2015; however, the trend has been decreasing since 2016, especially among outer-setting organizations. The top influencers from the three policy groups were the World Health Organization (WHO), the American Heart Association, and Tom Frieden. Simon Capewell and the WHO ranked the highest in activity; the World Action on Salt, Sugar, and Health and Action on Salt had the highest priority for dietary sodium content; General Mills and Tom Frieden had the highest originality; and WHO, Harvard University School of Medicine, and Tom Frieden received the highest popularity. Outer-setting organizations tend to interact with more users in the network compared to inner-setting organizations and individuals, while inner-setting organizations tend to receive more engagements from other users in the network than the other two groups. Monthly patterns showed a significant peak in the number of sodium posts in March compared with other months.

**Conclusions:**

Despite the increased use of social media, recent trends of sodium intake education on social media are decreasing and the priority of sodium among other topics is low. To improve policy implementation effectiveness and meet recommended dietary targets, there is an increasing need for health leaders to consistently and collectively advocate for sodium intake reduction on social media.

## Introduction

The excessive sodium content in most diets remains a significant challenge to global public health [1]. The World Health Organization (WHO) has estimated the mean global sodium intake to be 4310 mg per day, more than double the WHO recommendation of <2000 mg per day [1]. Excessive sodium intake is an established cause of raised blood pressure and elevated risk of cardiovascular disease (CVD) [1,2]. The Global Burden of Disease study identified a high-sodium diet as the leading dietary risk factor, with an estimated 3 million deaths attributable to it annually [3,4]. Unlike nonmodifiable risk factors, such as age and family history, sodium intake has been documented as a modifiable risk factor for CVD [5]. Interventions to reduce sodium intake are highly cost-effective in both developed and developing countries [6-9]. Specifically, a US study showed that a 10% average reduction in sodium intake, a reduction level similar to successful efforts in the United Kingdom, could prevent over 510,000 strokes and 480,000 myocardial infarctions in American adults [2].

Despite the release of several dietary guidelines, recent studies have observed no significant decline in sodium intake among American adults, and adherence to these guidelines is poor [10,11]. Many individuals are unaware of their salt intake or the effect of this intake on their long-term health, creating an increasing concern for and a need to raise awareness of sodium overconsumption [12]. Health literacy, defined as “the degree to which individuals have the capacity to obtain, process, and understand basic health information and services needed to make appropriate health decisions,” has been shown to relate to disparities in the use of health care services and consequently impact individual health outcomes [13-15]. A key venue for increasing health literacy is for health care professionals and health systems to provide access to health information [16]. Health systems can leverage channels for information dissemination to inform individuals about the negative health impacts of sodium overconsumption. As traditional information dissemination venues such as radio broadcasting, television, newspapers, and magazines adapt to the digital landscape, there is a need for increased research on digital means of information dissemination.

The use of social media for health education and promotion can play a key role in informing individuals on important health topics. Among several social media platforms, X (formerly Twitter) has emerged as a popular platform, with 70% of American adults using it as a source of news [17]. X has been widely used for health literacy promotion, infoveillance of health-related content, analyzing audience reach, and identifying stakeholder characteristics [17-19]. There is a need for an accessible tool that can quantify the impact of information dissemination on social media platforms and explain its driving factors. Mao et al [20] proposed a framework for measuring topic-specific influence on X, which quantifies the overall influence of each user by decomposing it into four dimensions: activity, popularity, originality, and priority. This framework was illustrated in a case study of dietary sodium–related posts [20]; however, the analysis was only conducted for 16 stakeholders, which may not have been representative of the health field landscape and provided limited insights for policy implementation.

This study aims to provide a more comprehensive understanding of the trends of dietary sodium–related information dissemination on X among stakeholders in the policy field from 2006 to 2022. We borrow Moulton and Sandfort’s [21] concept of policy fields in identifying sodium policy–related stakeholders on X. We define a policy field as a bounded network among organizations and individuals carrying out substantive policy making and information dissemination on a specific topic, and in this study, we focus on the topic of sodium reduction. We placed X users into one of three policy groups based on their role in the policy field: inner-setting organizations, such as public agencies that are directly involved in policy making; outer-setting organizations, such as research organizations that provide scientific evidence to facilitate policy making; and individuals, such as policy entrepreneurs, who may act across both the inner and outer settings. We analyzed the in-network interactions among users to identify the top influencers in different groups. After applying the 4D framework of Mao et al [20] to measure topic-specific influence, group-level and temporal trends of influence were investigated to understand the health leaders’ dissemination efforts on sodium intake reduction.

## Methods

### Overview

To understand the trends of dietary sodium–related information dissemination, we identified health domain users with a scope of work related to dietary sodium. Users were positioned into one of the 3 policy groups. All posts from these users from 2006 to 2022 were retrieved. We used social network analysis to identify the top influencers within each policy group and computed the 4D measures of influence for the top influencers. These results were used to reveal user-level, group-level, and temporal patterns of sodium-related influence.

### Account Selection

We took a 2-step approach, starting with a knowledge-informed “macrostructure” to identify influencers and then refined the selection using data-informed “micro behavior” analysis, as depicted in Multimedia Appendix 1. In this context, the macrostructure was outlined by 10 functional categories spanning public agencies, research and evaluation organizations, international organizations and initiatives, nongovernmental organizations, professional associations, policy entrepreneurs, philanthropies, scholars, food manufacturers, and advocacy organizations. We identified candidate influencers under each category by collecting a list of relevant organizations based on our domain knowledge, a list of experts from the New York City (NYC) Health Consensus Statement on Sodium [22], and top-ranked universities from US News regarding the top public health programs [23]. We then refined our list based on data, checking for whether these organizations and individuals had active accounts on X, posted any public health or sodium-related content, and what other accounts they interacted with in their sodium-related posts. This approach for account selection allowed us to comprehensively cover relevant stakeholders in dietary sodium reduction and ensure representatives from each category are included. In total, 78 X accounts were identified that encompassed domestic and international organizations across the 10 categories (see the full list in Multimedia Appendix 2).

### Account Categorizations

We conceptualize all X accounts as actors in policy fields, interacting with each other to carry out a substantive policy or program. Based on each organization or individual’s role in policy implementation, we categorized the corresponding X handle into one of three policy groups as either an inner-setting organization, an outer-setting organization, or an individual (Multimedia Appendix 3). We conceptualize the inner setting as a setting within the policy field in which policies are actively created. We define outer-setting organizations as organizations outside of the direct policy-making setting that still influence policy. We define individuals as actors with the potential to influence policy.

### Data Collection

All posts from the 78 accounts from X’s conception in March 2006 through May 31, 2022, were retrieved using the Academic Research licensed X (Twitter) Full-Archive Application Programming Interface V2. Post data contained the post ID, author username, post creation date and time, language of the post, post text, public metrics (count of reposts, replies, likes, and quotes), hashtags, whether the post was a repost, and mentions. Definitions of these terminologies can be found in X Glossary [24].

### Data Preprocessing

To determine which posts were related to dietary sodium, filtering was performed based on keyword criteria to label dietary sodium–related posts. First, English expressions containing “salt” with their meanings rooted in cultural or historical contexts that do not refer to dietary sodium were removed from the posts. Examples of slang terms used include “salt into the wound” or “salt of the earth.” Then, predefined sodium-related keywords and combinations with food-related keywords were used to flag dietary sodium content. Posts not containing keywords but using hashtags related to dietary sodium were also labeled as dietary sodium related. The full list of English expressions, keywords, and hashtags can be found in Multimedia Appendix 4. Note that only English posts were considered, as indicated in the language attribute of the post data.

### Network Analysis

Next, we used a data-driven approach to rank the initial sample of accounts. This sample can be seen as a community of stakeholders with a scope of work related to dietary sodium. We identified the most important accounts based on interactions within the community. First, we constructed a directed graph in which nodes represent users and edges are weighted based on the number of dietary sodium–related engagements from one user to another. User interactions on X included (1) a post mentioning another user, (2) a post quoting another user’s post, (3) a repost of another user’s post, and (4) a reply to another user’s post. We then used PageRank [25] to measure the in-network importance of each user based on sodium-related interactions. PageRank was originally proposed to assess the importance of web pages in the context of search engine results, with the underlying assumption that more important websites are likely to receive more and higher quality (ie, from other websites considered as important) links from other websites. These dynamics naturally translate to the X environment. In this context, PageRank scores can be interpreted as the likelihood that a person randomly browsing X will arrive at a user’s profile. We conducted the social network analysis using the networkx package [26]. We then selected the same number of top accounts from each policy group based on their PageRank scores to create an equally distributed sample for group-level analysis.

### Influence Analysis

The 4D framework of influence [20] was applied to measure the sodium-specific influence of each X account. Denote N as the number of posts, R as the number of reposts, P as the number of public engagements, and superscript ^t^ as dietary sodium specific. Influence is decomposed into four dimensions: (1) activity, which measures the level of participation in X, (2) priority of the topic within the scope of work of the user, (3) originality, which represents the proportion of posts that involve the creation of original content, and (4) popularity, which quantifies the average public attention received per post. A summary of the four measures and their equations is presented in Table 1. The overall measure of influence can be computed as:

Influence^t^ = Activity × Priority × Originality^t^ × Popularity^t^,

which can be shown to be equivalent to the total public engagements received from all topic-specific posts.

**Table 1:** 4D framework of influence.

Dimension	Description	Measure
Activity	Level of participation	N
Priority	Relative importance of a given topic within the scope of work	N^t^/N
Originality	Proportion of posts that required creation of original content	(N^t^ – R^t^)/N^t^
Popularity	Average number of public engagements received per (content-creation) post	(P^t^_like_ + P^t^_reply_ + P^t^_quote_ + P^t^_repost_) / (N^t^ – R^t^)

### Ethical Considerations

Since the data used in this study are publicly available on X with minimal risks to human participants, this study was deemed exempt from review by the University of Washington’s institutional review board (#18401). The data used in this study were obtained with explicit consent from X for research purposes. As users of X data, we only accessed data that is publicly available.

## Results

### Overall Trends

**Figure 1:**
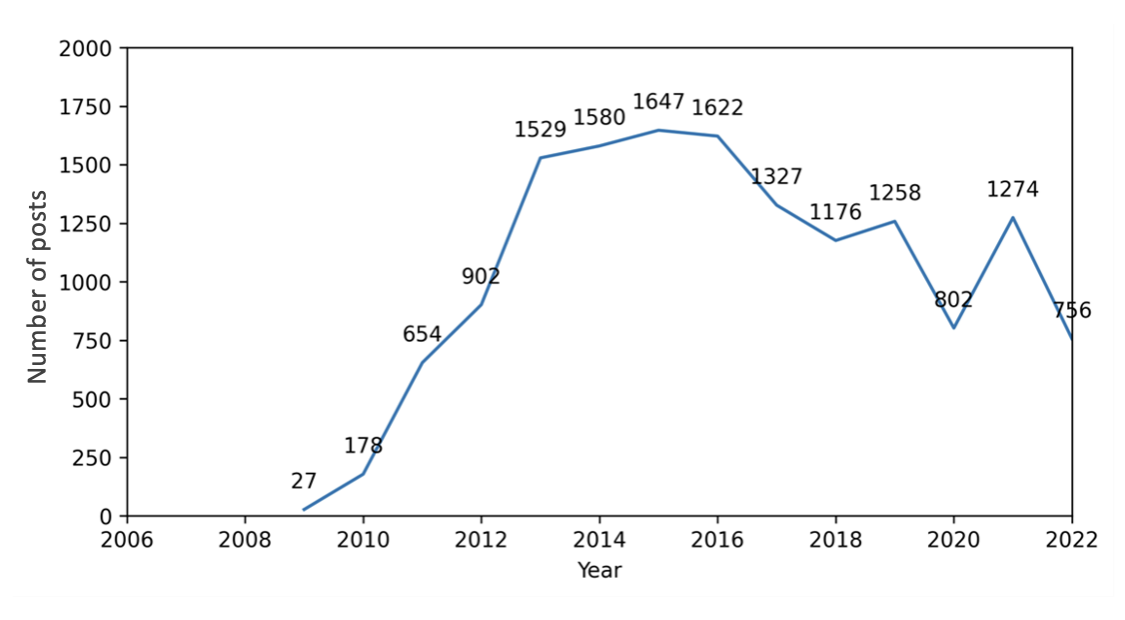
Total number of dietary sodium posts from all X accounts from March 2006 to May 2022.

Of the initial 78 users, 6 accounts did not have any dietary sodium–related posts (Departmentofh14, WHOSTP, SJPHonline, UCLeHealth, ChanZuckerberg, and Kcferdmd). The remaining 72 accounts totaled 1,099,605 posts and 14,732 dietary sodium–related posts. The number of dietary sodium–related posts from these accounts significantly increased from 2010 to 2015, increasing from 178 dietary sodium posts in 2010 to 1647 dietary sodium posts in 2015 (Figure 1). However, the number of dietary sodium posts began to decrease after 2016, with small increases in 2019 and 2021.

### In-Network User Interactions

**Figure 2:**
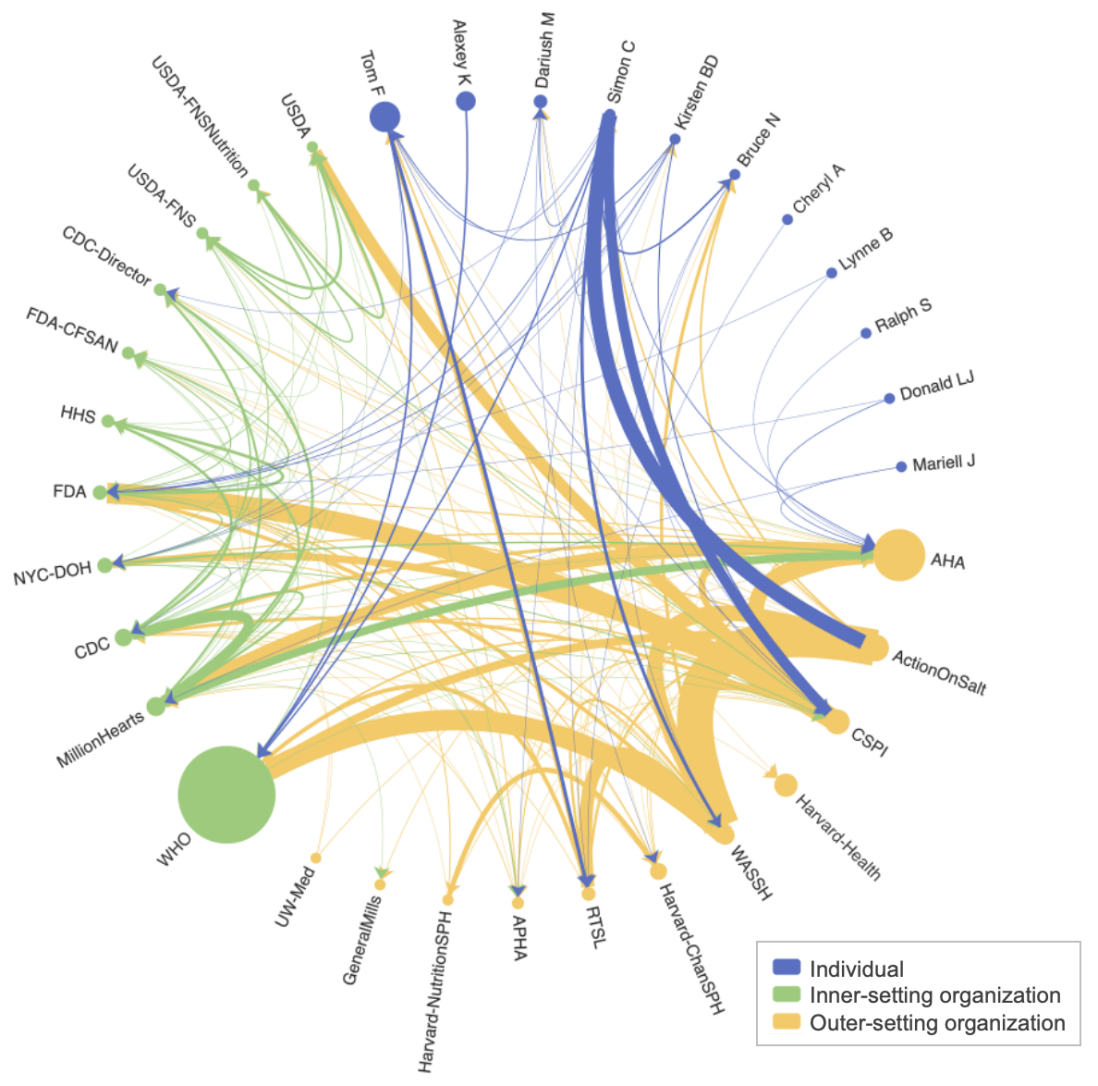
Dietary sodium–related engagements among top users from each policy group selected based on in-network importance. Each node represents an X user, with the color of the node indicating the policy group of the users and the size of the node representing the overall influence score of the user (ie, the larger the node, the more dietary sodium influence the user had). The arrows indicate the direction of the engagements, and the thickness of the arrows represents the number of engagements between users (ie, the thicker the line, the more engagements from one user to the other user). For an interactive figure, refer to [27]. AHA: American Heart Association; APHA: American Public Health Association; CDC: Centers for Disease Control and Prevention; CFSAN: Center for Food Safety and Applied Nutrition; CSPI: Center for Science in the Public Interest; FDA: Food and Drug Administration; HHS: Department of Health and Human Services; NYC-DOH: New York City Department of Health and Mental Hygiene; RTSL: Resolve to Save Lives; USDA: US Department of Agriculture; USDA-FNS: US Department of Agriculture Food and Nutrition Service; WASSH: World Action on Salt, Sugar, and Health; WHO: World Health Organization.

The top 11 users with the largest PageRank scores from each policy group were selected for a more detailed influence analysis. Figure 2 [27] shows the dietary sodium–related interactions among the top influencers in each policy group. X accounts from the WHO, the American Heart Association (AHA), and Tom Frieden had the greatest overall influence at each policy level. The WHO had significantly higher dietary sodium–related influence than other users, with an overall influence measure of 55,593, while the AHA and Tom Frieden had an overall influence of 26,395 and 12,672, respectively.

Frequent interactions were observed within the top influencers (Figure 2 [27]). The WHO received a high volume of engagements from users of all three policy groups but did not frequently engage with other users in the network. The strongest amount of dietary sodium–related engagement the WHO received within the network was from the World Action on Salt, Sugar, and Health (WASSH), a global group founded with the mission to reduce salt intake to the WHO-recommended levels. WASSH not only actively interacted with large organizations including WHO but also had reciprocal interactions with similar initiatives including Action on Salt, as well as with global public health initiatives such as Resolve to Save Lives (RTSL). The Center for Science in the Public Interest (CSPI) is another example of a highly active organization that engaged with many other national organizations, including AHA, the US Department of Agriculture (USDA), the US Food and Drug Administration (FDA), the NYC Department of Health and Mental Hygiene (NYC-DOH), and the Centers for Disease Control and Prevention (CDC). Frequent mutual interactions were also observed between partnered entities such as those between the AHA and Million Hearts, a national initiative co-led by the CDC and the Centers for Medicare and Medicaid Services with the mission to prevent CVD; and those between RTSL and Tom Frieden.

Outer-setting organizations tend to engage with more users in the network showing broader in-network outreach efforts. Specifically, outer-setting organizations engaged with 8.4 (SD 6.4) other users within the network on average, followed by inner-setting organization users engaged with 5.3 (SD 3.0) other users on average, and individual users with 3.4 (SD 3.5) other users. The CSPI, WASSH, and Action on Salt had the broadest sodium-related in-network outreach, with 18, 17, and 15 other users, respectively. On the other hand, inner-setting organizations received engagements from more users in the network from 9.7 (SD 5.2) other users on average, compared to outer-setting organizations from 5.7 (SD 5.3) users on average, and individuals from 1.5 (SD 2.1) other users on average. FDA and CDC received engagements from 21 and 17 other users in the network. Simon Capewell was the most active individual user, engaging with 13 other users in the network, while Tom Frieden received the most engagement from other users. Multimedia Appendix 5 shows a summary of these results.

### Annual Trend of Influence Across Policy Groups

**Figure 3:**
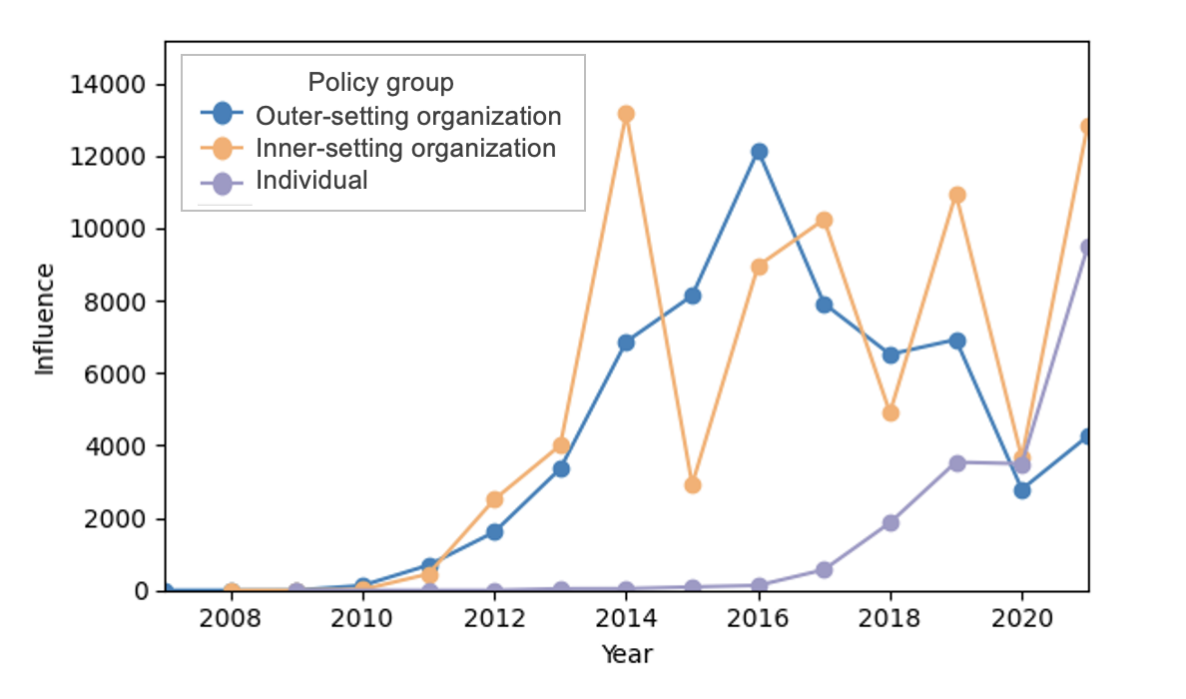
Annual trends of total dietary sodium–related influence of top users from the three policy groups. Years without 12 months of data were excluded (years 2006 and 2022) for a fair comparison.

The annual trend of the total dietary sodium–related influence of top users in each policy group is shown in Figure 3. Inner-setting and outer-setting organizations had greater influence than individual users overall. The sodium-related influence of inner-setting organizations fluctuated considerably across all years. Alternating spikes and sudden decreases in dietary sodium influence occurred roughly every other year, varying by more than 60% in some years. In contrast, outer-setting organizations experienced a steady increase in dietary sodium–related influence from the launch of X in 2006 through 2016, followed by a decline thereafter. A dip in dietary sodium influence was observed in 2020 for both inner-setting and outer-setting organizations although not for individual users. The influence of individual users has been increasing over the years, with a significant spike in 2021.

**Figure 4:**
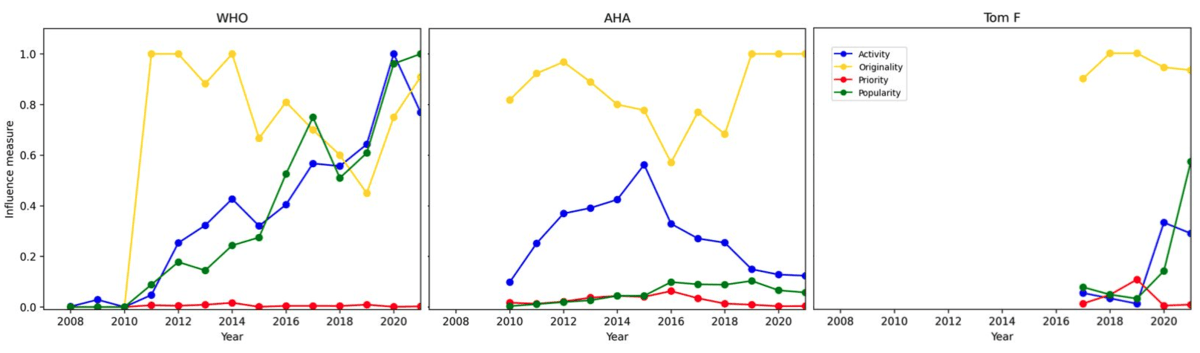
Top influencers in each policy group and their four dimensions of influence. Years without 12 months of data were excluded (years 2006 and 2022) for a fair comparison. The three panels in the figure represent the top influencers in each policy group: the WHO for inner-setting organizations, the AHA for outer-setting organizations, and Tom Frieden for individual users. Each plot displays a yearly comparison of the four influence dimensions. Originality and popularity ranged from 0 to 1. Activity ranged from 168 to 11,612. Popularity ranged from 0 to 528. For visualization purposes, activity and popularity were scaled to be between 0 and 1. AHA: American Heart Association; WHO: World Health Organization.

The WHO emerged as the leading influencer among inner-setting organizations, the AHA led among outer-setting organizations, and Tom Frieden stood out as the top influencer among individual users. The WHO had a steadily increasing trend of posting volume since joining X in 2008 and received a similar increase in average public attention per original post (Figure 4, left panel). A significant increase in activity and popularity was observed in 2020, which was coincident with the COVID-19 pandemic. Despite having one of the largest posting volumes in this sample, the WHO’s priority for dietary sodium–related posts remained very low across all years, given WHO’s large scope of work. The WHO did not post any dietary sodium–related posts until 2011 and consequently, priority was zero in the first 3 years prior and remained near zero across later years. In terms of originality, the WHO posted the highest percentage of original sodium-related content in 2013, coinciding with the Member States' adoption of the global action plan for the prevention and control of noncommunicable diseases [28]. From 2014 to 2019, the WHO steadily relied more on reposts, but originality began increasing again in 2020 as the target year for achieving a 30% relative reduction in population sodium intake approached

The AHA had increasing posting activity from the creation of their account in 2010 through 2015 but the posting volume started decreasing following that year (Figure 4, middle panel). Their priority of dietary sodium posts was comparatively higher than the WHO for many years, especially from 2013 through 2017, which is expected given that the AHA’s scope of work in CVD is closely related to sodium intake. The originality of their dietary sodium posts was in a similar range as the WHO in earlier years, reaching 100% originality starting in 2019 indicating the AHA’s efforts in content creation for sodium-related topics.

Despite being one of the newest to join the social media platform, Tom Frieden was by far the top influencer among the individual users (Figure 4, right panel). His posting activity level was typically lower than WHO and AHA. Tom Frieden had significantly higher priority placed on dietary sodium–related topics in 2018 and 2019 than the other top influencers and tended to prefer posting original content rather than reposts. We present a more detailed case study for Tom Frieden later in this section.

### Leaders in Four Dimensions of Influence

**Figure 5:**
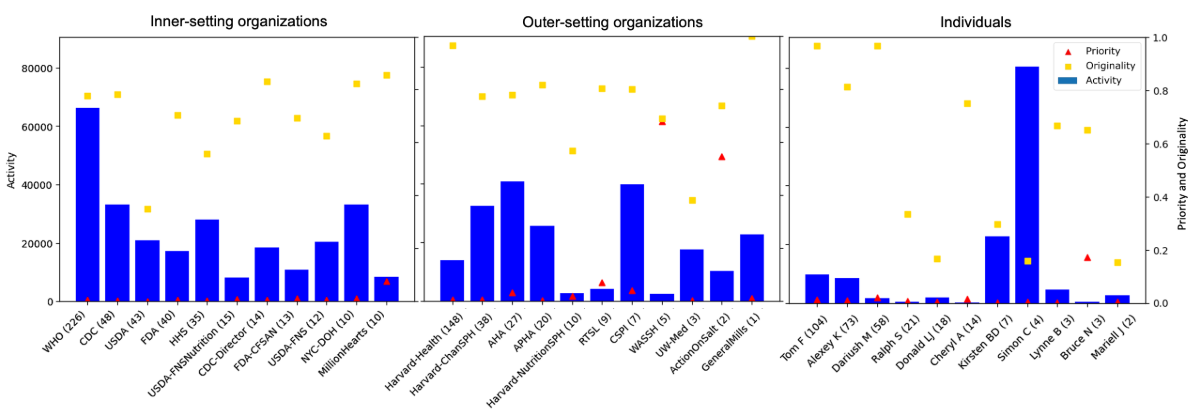
Comparison of activity, priority, originality, and popularity across users in each policy group. Activity is displayed in the blue bars and measured based on the left y-axis. Priority and originality are displayed as the red triangles and yellow boxes, respectively, and measured based on the right y-axis. Popularity is displayed in parentheses next to the X handle names. Within each policy group, users are arranged by popularity. AHA: American Heart Association; APHA: American Public Health Association; CDC: Centers for Disease Control and Prevention; CFSAN: Center for Food Safety and Applied Nutrition; CSPI: Center for Science in the Public Interest; FDA: Food and Drug Administration; HHS: Department of Health and Human Services; NYC-DOH: New York City Department of Health and Mental Hygiene; RTSL: Resolve to Save Lives; USDA: US Department of Agriculture; USDA-FNS: US Department of Agriculture Food and Nutrition Service; WASSH: World Action on Salt, Sugar, and Health; WHO: World Health Organization.

A comparison of the four influence measures across the users in each policy group across all years is shown in Figure 5. Overall, outer-setting organizations tend to have higher activity levels than inner-setting organizations or individuals. Among inner-setting organizations, the WHO demonstrated the highest level of posting activity, nearly doubling that of the next most active organization. This further underscores the WHO’s global leadership in sodium reduction efforts. Despite most individual users having relatively low activity levels as compared to organizations, Simon Capewell was an outlier who posted the largest number of posts in this sample, with nearly 80,000 posts since conception, more than the total number of posts by the WHO. It is worth noting that roughly 80% of Simon Capewell’s posts were reposts, indicating that the main role of his account is a disseminator rather than a content creator.

The presence of dietary sodium posts out of total posts (measured by priority) is very low among the overall efforts of these users regardless of their policy group. Among outer-setting organizations, the WASSH and Action on Salt had the highest priority for dietary sodium content, with over half of their contents related to dietary sodium topics. The individual user Bruce Neal, who is a UK-trained physician and currently executive director at the George Institute for Global Health Australia, received priority scores of approximately 20%, ranking highest among inner-setting organizations. The US-based initiative Million Hearts had a priority score of around 10%. Following them are outer-setting organizations RTSL, the AHA, and CSPI. The remaining users had less than 2% of their posts related to dietary sodium.

In terms of popularity, the WHO also ranked the highest across all policy groups. Notably, larger posting volume is not always associated with higher public attention per post. Tom Frieden and Harvard School of Medicine (Harvard-Health) are among the highest top three in the sample despite having low-mid level activity.

Inner-setting and outer-setting organizations had relatively similar levels of originality. General Mills, Harvard-Health, Tom Frieden, and Dariush Mozaffarian had the highest levels of originality with over 95% of their sodium-related posting efforts dedicated to original content. In contrast, the USDA and UW School of Medicine (UW-Med) had the lowest originality among the inner-setting and outer-setting organizations with over 60% of their sodium-related posts being reposts. There is more variability of originality within the individual category. About half of individual users focus more on content creation and the other half focus more on content dissemination.

### Monthly Posting Patterns

The total number of dietary sodium–related posts in each month across all policy-type users from 2007 through 2021 is shown in Table 2. A noticeable peak of posting volume is observed in March. The other months tend to stay relatively consistent with the number of dietary sodium posts, averaging about 857 dietary sodium posts per month, over four times less than the volume in March.

**Table 2:** Monthly posting patterns of dietary sodium–related posts from 2007 to 2021. Years without 12 months of data were excluded (years 2006 and 2022) for a fair comparison.

Month	Number of dietary sodium–related posts
January	792
February	857
March	3432
April	930
May	801
June	971
July	989
August	795
September	1161
October	690
November	761
December	678

### A Notable Example: Tom Frieden

**Figure 6:**
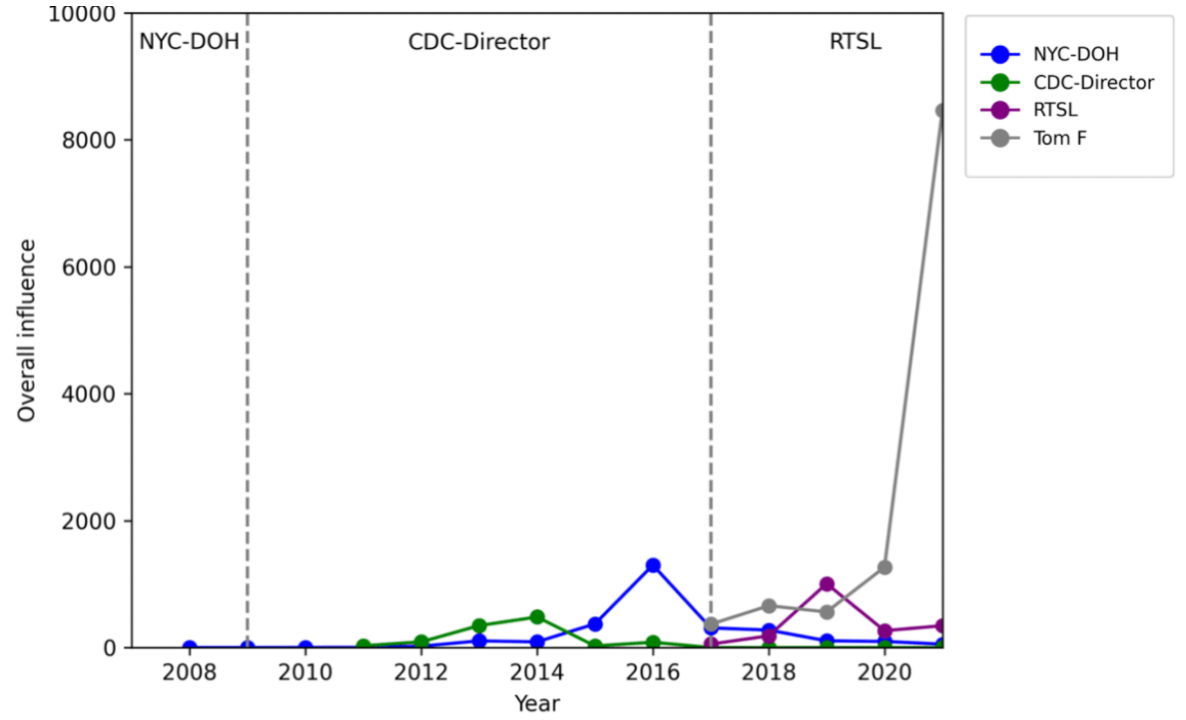
An example of a policy entrepreneur: Tom Frieden. The line plot visualizes the influence evolution of Tom Frieden’s personal account and the three accounts of the organizations he served at. Partitions provided to indicate when Tom Frieden worked for each group. CDC: Centers for Disease Control and Prevention; NYC-DOH: NYC Department of Health and Mental Hygiene; RTSL: Resolve to Save Lives.

To provide a more specific example of analysis, we consider Tom Frieden, a physician and notable public figure in public health who has played numerous roles in both the inner-setting and outer-setting organizations. As shown in Figure 2 [27], Tom Frieden was not only the top-ranked individual user in terms of overall influence of dietary sodium content but also had close relationships with three other accounts identified in our analyses as being influential (NYC-DOH, CDC-Director, RTSL). He serves as President and Chief Executive Officer at RTSL starting in 2017, and previously was Commissioner at the NYC-DOH from 2002 to 2009 and Director of the CDC from 2009 to 2017 [29]. Tom Frieden created his personal X account in 2017. The dietary sodium–related influence of the various X accounts that have been associated with Tom Frieden is displayed in Figure 6 with partitions provided to indicate when he worked for each organization.

Having held leadership roles in multiple health organizations, Tom Frieden’s personal account had a higher overall influence than the accounts of the organizations he served at. Tom Frieden’s personal account reached the greatest dietary sodium–related influence in 2021, over six times that of the next highest influence account, which was that of the NYC-DOH*,* in 2016. The NYC-DOH account did not have any dietary sodium posts from 2006 to 2009 when Tom Frieden was Commissioner. The CDC-Director account was created in 2011 when Tom Frieden was serving as Director at the CDC and had consistent growth in sodium-related influence from 2011 to 2014. However, the CDC-Director’s activity on dietary sodium topics dropped to zero after his departure from the CDC in 2017 and in the years after. The RTSL account was established when Tom Frieden joined RTSL in 2017, and its sodium-related influence has steadily grown since its creation, aligning with the sodium-focused priorities of Tom Frieden’s personal account. RTSL had a relatively higher dietary sodium influence with about 319 average public engagements each year, which may be partly attributed to Tom Frieden’s frequent interactions with RTSL to increase its visibility. Given the creation of both the CDC-Director and RTSL during his roles at the respective organizations, as well as the lack of dietary sodium influence with the CDC-Director after he left the CDC, Tom Frieden appears to have catalyzed the information dissemination of dietary sodium topics in these organizations.

## Discussion

### Overall Trends

This study leveraged the 4D influence framework and the concept of policy fields to analyze health leaders’ efforts in disseminating dietary sodium–related content on X in the past 16 years. Overall, the presence of sodium intake–related content is very low among the overall dissemination efforts of health leaders across all policy groups, except for initiatives specialized in sodium consumption such as WASSH and Action on Salt. Initially, dissemination efforts about dietary sodium content increased after the WHO’s announcement of sodium reduction targets in 2013 [28]. Two years later, the USDA released Dietary Guidelines for Americans [30], which could explain the rise of dietary sodium intake discussion on social media and the spike in outer-setting and inner-setting organizations’ sodium-related influence in 2016. However, the overall dissemination efforts on sodium intake have been decreasing since then. A local peak was observed in 2021, which may be attributed to the National Academy of Sciences, Engineering, and Medicine’s establishment of a chronic disease–specific recommendation for dietary sodium in 2019 [31] followed by the USDA 2020 Dietary Guidelines for Americans [30] attracting some attention in sodium reduction. The sodium-related influence of outer-setting organizations, in particular, has largely decreased from 2020 to 2022, which is possible due to these organizations redirecting efforts to other health topics related to the COVID-19 pandemic. In contrast, individual users, particularly Tom Frieden, gained significantly more public attention during the pandemic and continued advocating for the prevention of CVD. Inner-setting organizations exhibit a sporadic pattern of influence on sodium-related topics with large fluctuations year over year. This could be related to the inconsistency of their efforts in addressing the topic of dietary sodium intake or related to external factors that would impact the engagement they received on their content.

### Comparison Across Policy Groups

Comparing the three policy groups, inner-setting and outer-setting organizations had generally greater overall influence as opposed to individual users. This may be because organizations typically constitute larger groups with more access to resources for health advocacy and social media dissemination. Individual users likely do not have comparable resources but may instead focus on increasing the visibility of their posts on social media through strategies such as using hashtags and interacting with larger organizations or increasing their activity through less time-consuming reposting. It is worth noting that the top two individual influencers, Tom Frieden and Alexey Kulikov, both serve or served in leadership positions in inner-setting organizations.

### In-Network User Interactions

A significant volume of interactions was observed among the top influencers. A potential reason behind these interactions is the collaboration of efforts to reduce dietary sodium consumption. For example, RTSL and WASSH were observed to have reciprocal interactions, possibly due to the funding provided by RTSL for a salt reduction toolkit led by WASSH [32]. Another reason the organizations interact is to hold each other accountable to drive positive change. We observed that CSPI actively engaged with the FDA. A recent press release noted that the CSPI has been recommending the FDA to decrease the sodium in foods since 1978 and continues to advocate for sodium reduction, including filing petitions for the FDA to better regulate sodium in processed food [33]. Outer-setting organizations tend to interact with more users in the network compared to inner-setting organizations and individuals, while inner-setting organizations tend to receive more engagements from other users in the network than the other two groups. Leveraging the professional authority can enhance the legitimacy of policy dissemination. For instance, WASSH and Action on Salt engaged the WHO as an effective strategy to promote international sodium reduction guidelines and scientific evidence.

### Leaders in Four Dimensions of Influence

The top influencers from inner-setting organizations, outer-setting organizations, and individual policy groups were the WHO, AHA, and Tom Frieden, respectively. As a large global organization, the WHO had a steadily increasing presence and popularity on social media over the years. The WHO is the most widely engaged health organization among all health leaders, which reinforces its influential position as the goal-setting organization for sodium reduction. However, the priority of advocating for sodium intake reduction is very low (<1% of total posts) in the WHO’s broad scope of work. Expectedly, the AHA had relatively higher priority for sodium content than the WHO given its goal of fighting heart disease through education. The AHA’s priority for advocating for sodium intake reduction peaked in 2016, likely due to the release of Dietary Guidelines for Americans [30]. However, less than 2% of their annual posts were related to sodium since 2018. Having held leadership roles in both inner-setting and outer-setting organizations, Tom Frieden has been a prominent health policy entrepreneur and social media influencer. Since 2019, Tom Frieden’s personal account has posted an annual volume of posts similar to large organizations such as the AHA. He peaked in priority for sodium content in 2019 since the creation of RTSL in 2017, periodically interacting with RTSL's account through his personal account, and continued advocating for combating high blood pressure and CVD but with a lower portion of posts discussing sodium intake in 2019. Tom Frieden is a notable example of a driver of health education through social media, not only through his personal account but also by promoting the accounts of organizations he served at. Tom Frieden is likely a common factor contributing to the rise in influence of the CDC-Director and RTSL accounts during his service at those organizations.

Originality, which indicates a preference for content creation, is an important but not sole driver of influence. While both inner-setting and outer-setting organizations have similar levels of originality, with about one-third of posts being reposts, there is more variability of behavior among individual users. Some individual users are original content creators (eg, Tom Frieden and Dariush Mozaffarian), who focus on sharing their expertise in health and sodium consumption, and others are content disseminators (eg, Simon Capewell, Mariell Jessup, and Donald M Lloyd-Jones), who actively disseminate the content created by other authorities. While content creators may attract public attention by providing their unique expertise, disseminators have the advantage of time efficiency which allows them to publish a large volume of posts with less effort. A notable example is Simon Capewell, who had the highest level of activity in the sample despite being an individual user.

### Monthly Posting Patterns

Analyzing the monthly trends of posts across all years reveals a significant peak of dietary sodium–related posts in March. This is likely because National Nutrition Month (#NationalNutritionMonth), as well as World Salt Awareness Week (#SaltAwarenessWeek, #WorldSaltAwarenessWeek) typically occur in March, both of which can lead to an increase in sodium-related discussions on social media [34,35]. Such an increase in attention for sodium consumption was not observed for other sodium-related events such as Stroke Awareness Month (#StrokeAwarenessMonth) and Hypertension Awareness Month (#HighBloodPressureMonth) in May or the National Heart Month (#NationalHeartMonth) in February.

Factors that contributed to a higher sodium intake include knowledge, attitude, and habits, which can be modified by education [36,37]. Given the increasing use of social media by the general public, there is an increasing need for health leaders to advocate for sodium intake reduction and use social media as an important platform for health education. Theories of implementation effectiveness convey the importance of (1) building a strong implementation climate, which refers to the targeted user’s shared perceptions about the impact of the policy, and (2) delivering the fit of that policy to the targeted user’s values [38,39]. Additionally, fostering shared beliefs and collection action among interdependent stakeholders is critical for successful implementation [39,40]. Social network advertising has become a key component of strategic communication and audience engagement [41]. Thus, it is crucial for health leaders to collectively and consistently advocate for the recommended dietary guidelines of sodium intake and the long-term health risks of current sodium intake levels.

### Comparison With Prior Work

To the best of our knowledge, the first work to analyze the public influence of sodium-related posts of health organizations and leaders is Mao et al [20]. We include a broader range of stakeholders selected from 10 different categories to provide results that are more representative of the public health landscape. By including a larger sample, our analysis found influencers who displayed relatively high priority (Million Hearts*,* Bruce Neal, and RTSL), popularity (Harvard-Health), originality (Dariush Mozaffarian, Harvard-Health, and General Mills), and activity (Department of Health and Human Services, NYC-DOH, and American Public Health Association), which were not included in Mao et al’s analysis. In terms of the accounts analyzed by both studies, our analysis found similar patterns. Note that the top influencers found by our analysis account for a fifth dimension of influence which is the in-network importance. As a result, the Food and Agriculture Organization of the United Nations was a top influencer in the study by Mao et al [20] but not considered a top influencer in our PageRank analysis. In terms of study objectives, Mao et al [20] focused on evaluating the dissemination behavior of stakeholders to reveal their strengths and weaknesses. In contrast, we aim to understand stakeholders’ role in policy implementation and its relationship to their health information dissemination behaviors. This was accomplished by positioning stakeholders within one of the three policy groups, analyzing their interactions via network analysis, and comparing the patterns of outer-setting organizations, inner-setting organizations, and individuals at a policy group level. Our results reveal that these three types of users exhibited different temporal evolution of sodium-related influence possibly due to their distinct reactions to national and international policy events such as the WHO’s sodium reduction target announcement, the USDA’s release of dietary guidelines.

We applied Moulton and Sandfort’s [21] concept of policy fields to categorize the users into three policy groups, though there exist other frameworks. Cruden et al [42] defined five categories of policy actors: (1) policy developers, who shape the vision or intention of a policy; (2) policy disseminators, who communicate the policy with relevant stakeholders; (3) policy implementers, who usually have decision-making authority in their organization and lead the adoption of the new policy into their practice settings; (4) policy influencers, who can be formal or informal actors that impact which evidence is used in which ways during policy implementation; and (5) policy enforcers, who monitor, communicate, and support the adoption and implementation quality of the policy. While this categorization is more detailed, it is difficult to apply to our analysis because many times an organization or individual could fall into multiple categories of policy actors.

### Limitations

There are several limitations in this study. First, we used keyword-based criteria to determine whether a post was dietary sodium–related. Although we intended to curate a comprehensive list of salt-related expressions, hashtags, and food-related synonyms, this labeling method may still incorrectly classify some posts. Using more advanced natural language processing techniques is a possible improvement direction. Second, our account selection focused on organizations and individuals with the scope of work in public health and dietary sodium–related posts and may exclude unconventional users with potential impact on this topic. Third, our analysis only focused on English posts, while some international organizations such as the WHO published a small portion of posts in other languages. Fourth, the 4D framework used in this study focused on measuring influence in terms of quantifiable engagements with social media content. Content analysis of individual posts is outside the scope of this work. Fifth, our analysis focuses on X and excludes other social media platforms where influencers might be active. Future research is warranted to continuously track the dietary-related sodium influence on social media after 2022 and extend the analysis to other nutritional policies on sugar and trans-fat.

### Conclusions

This study analyzed the dietary sodium–related dissemination efforts of national and international public health leaders on the social media platform X from 2006 to 2022. We used the 4D framework to measure the public influence of sodium-related posts [20] and applied Moulton and Sandfort’s [21] policy field concept to relate dissemination behaviors with the users’ role in policy implementation. Our analysis identified top influencers in sodium content dissemination (WHO, AHA, Tom Frieden, and others), analyzed in-network interactions among the health leaders, and contrasted the behavior of the inner-setting, outer-setting, and individual policy groups. Despite the increased use of social media over the last decade, the overall dissemination efforts in sodium intake education have been decreasing from 2016 to date, particularly for outer-setting organizations. The priority of sodium-related topics is very low among the health leaders across all policy groups except for specialized initiatives. Given the cost-effectiveness of sodium reduction on CVD prevention and the lack of awareness of the health risks of high sodium intake by the public, there is an increasing need for health leaders to consistently and collectively advocate for the reduction of sodium consumption on social media to achieve the recommended dietary targets.

## Appendix

Multimedia Appendix 1 A two-step approach for account selection leveraging knowledge-informed macro-structure and data-informed micro behaviors.

Multimedia Appendix 2 All accounts categorized by policy group.

Multimedia Appendix 3 Policy groups in information dissemination.

Multimedia Appendix 4 List of dietary sodium keywords, English expression terms excluded from tweets, dietary sodium-related hashtags, and food-related keywords.

Multimedia Appendix 5 Number of users to interact with within the network for each top influencer.

## Abbreviations

AHA: American Heart Association
CDC: Centers for Disease Control and Prevention
CSPI: Center for Science in the Public Interest
CVD: cardiovascular disease
FDA: US Food and Drug Administration
NYC-DOH: New York City Department of Health​ and Mental Hygiene
RTSL: Resolve to Save Lives
USDA: US Department of Agriculture
WASSH: World Action on Salt, Sugar, and Health
WHO: World Health Organization

## Data Availability

The datasets generated during or analyzed during this study are not publicly available due to X (Twitter)’s data sharing policies but are available for retrieval from X’s Full Archive Application Programming Interface on access approval from X [43].
