# Supplementary figures and images for: Influencers in Policy Fields on Social Media: Global Longitudinal Study of Dietary Sodium Reduction Posts, 2006-2022
Source: J Med Internet Res. 2024 Dec 30;26:e54506. doi: 10.2196/54506 (PMC11730221; doi:10.2196/54506)

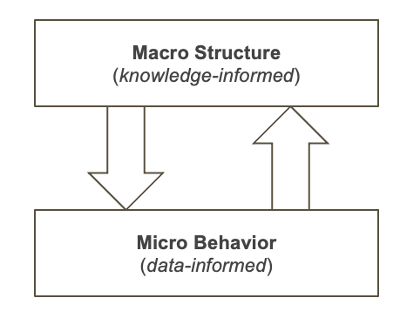

Supplement: Multimedia Appendix 1 [file jmir_v26i1e54506_app1.png]

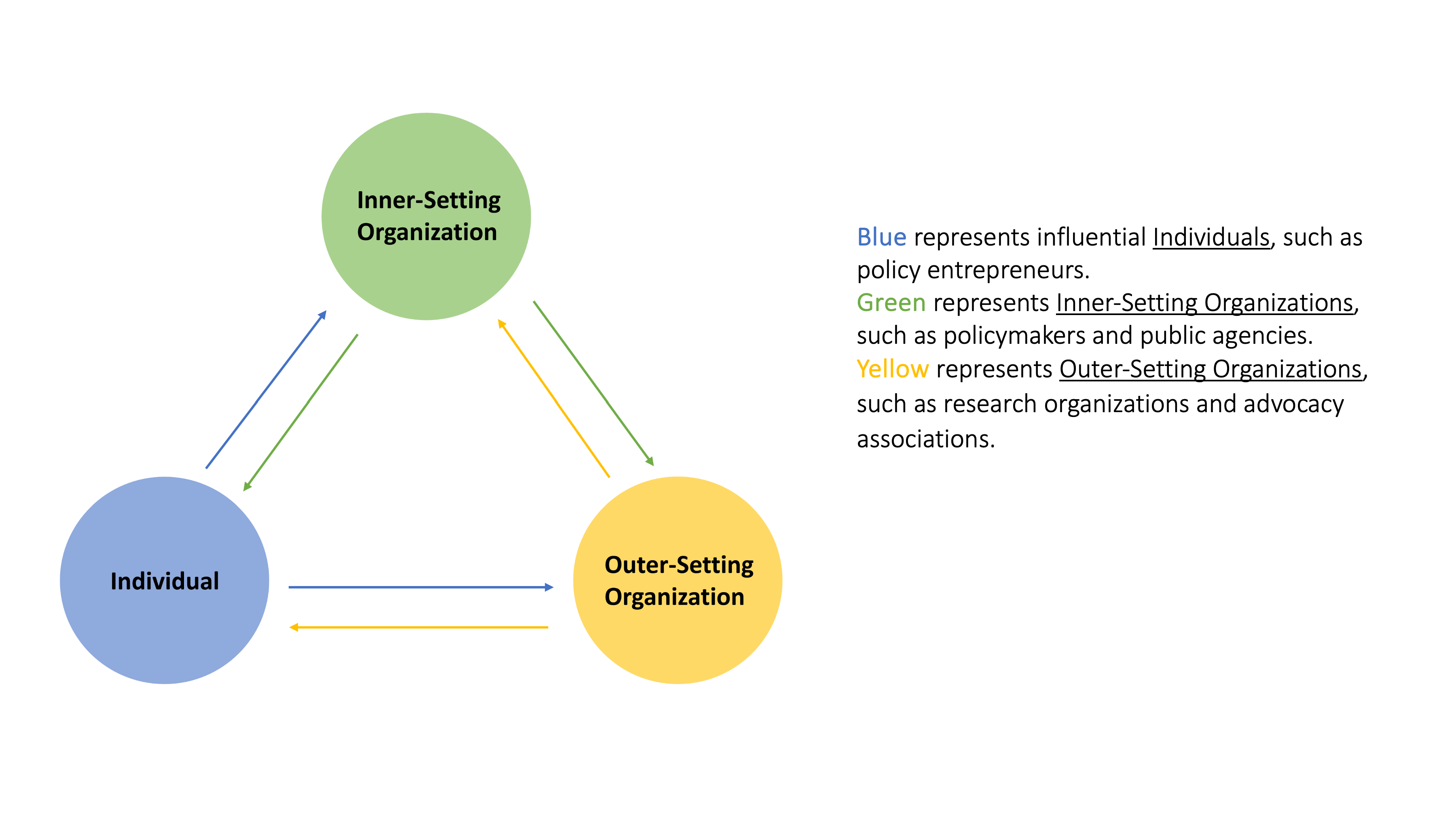

Supplement: Multimedia Appendix 3 [file jmir_v26i1e54506_app3.png]
