# Supplementary material for: Influencers in Policy Fields on Social Media: Global Longitudinal Study of Dietary Sodium Reduction Posts, 2006-2022
Source: J Med Internet Res. 2024 Dec 30;26:e54506. doi: 10.2196/54506 (PMC11730221; doi:10.2196/54506)
Supplement: Multimedia Appendix 4 [file jmir_v26i1e54506_app4.docx]

Dietary sodium keywords:

“sodium” OR “ salt ” or “#salt ” OR (“salts” OR “salty” OR “salted”) AND (“food” OR “diet” OR “health” OR “ eat” OR “ meal” OR “intake”) AND (at least one "food" or "eating" synonyms) OR (“lesssalt” OR “lowsalt” OR “highsalt” OR “saltawareness” OR “saltreduction)

List of English expressions excluded from Tweets:

'pinch of salt', 'dash of salt', 'worth their salt', 'worth his salt', 'worth her salt', 'worth my salt', 'worth your salt', 'grain of salt', 'attic salt', 'eat their salt', 'dose of the salts', 'salt into the wound', 'salt in a wound salt down', 'salt away', 'salt horse', 'salt of the earth', 'salt the books', 'salt-and-pepper', 'above the salt', 'back to the salt mines', 'below the salt', 'pound salt', 'old salt'

List of dietary sodium-related hashtags:

'switchthesalt', 'breakupwithsalt', 'escapethesalt', 'hiddensalt', 'noaddedsalt', 'nosaltplease', 'nycsalt', 'reducesalt', 'restrictsalt', 'saltintake', 'saltsubsitute', 'saltsubstitutes', 'saltsugfat', 'saltswitch', 'shakethesalthabit', 'sneakysalt', 'saltweek2018', 'stopsaltstopstroke', 'stopthehiddensalt', 'toomuchsalt', 'uksaltchallenge', 'unsalted', 'spotthesalt'

“food” synonyms

'bread', 'cooking', 'cuisine', 'drink', 'fare', 'foodstuff', 'meal', 'meat', 'snack', 'bite', 'board', 'chow', 'cookery', 'diet', 'eats', 'goodies', 'groceries', 'grub', 'handout', 'nutrition', 'ration', 'sustenance', 'table', 'entrée', 'fast food', 'home cooking', 'take out'

“eating” synonyms:

'chewing', 'consumption', 'gobbling', 'munching', 'nibbling', 'overindulgence', 'snacking', 'feasting on', 'feeding on', 'gorging on', 'having a meal', 'pigging out', 'stuffing oneself'

The “food” and “eating” synonyms were identified by consulting with the English Thesaurus (https://www.thesaurus.com). For each synonym that appeared for “food” and “eating”, Google was searched to identify if the synonym could appear with “salt” or “salty”. If a synonym did, the term was added to the keywords list.
